# Retinol binding protein 4 levels relate to the presence and severity of coronary artery disease

**DOI:** 10.5937/jomb0-28846

**Published:** 2021-09-03

**Authors:** Gokay Nar, Sara Cetin Sanlialp, Rukiye Nar

**Affiliations:** 1 Pamukkale University, Faculty of Medicine, Department of Cardiology, Denizli, Turkey; 2 Servergazi State Hospital, Department of Cardiology, Denizli, Turkey; 3 Pamukkale University, Faculty of Medicine, Department of Medical Biochemistry, Denizli, Turkey

**Keywords:** adipokine, acute coronary syndrome, retinol binding protein 4, CAD severity, adipokin, akutni koronarni sindrom, retinol vezujući protein 4, težina KAO

## Abstract

**Background:**

The previous studies have showed that serum retinol binding protein 4 (RBP4) levels increase in metabolic disorders which are closely associated with cardiovascular diseases (CVD). However, the human studies investigating the role of RBP4 in CVD are conflicted. Therefore, we aimed to evaluate the relationship between RBP4 with the presence and severity of coronary artery disease (CAD) in this study.

**Methods:**

55 patients with presenting acute coronary syndrome (ACS) and 43 control subjects who had various cardiovascular risk factors with normal coronary artery on coronary angiography were included in this study. The serum RBP4 concentrations were measured using ELISA method, clinically and anatomically score models were used to assess the severity of coronary lesion.

**Results:**

Serum RBP4 levels were significantly higher in patients with ACS compared to the without ACS (68.40 ± 47.94 mg/L vs. 49.46 ± 13.64 mg/L; p = 0.014). RBP4 was correlated with GENSINI and SYNTAX I score (r = 0.286 p = 0.034; r = 0.403 p = 0.002 respectively). However, there was no relationship between RBP4 and GRACE score.

**Conclusions:**

The serum RBP4 levels increase in patients with CAD and its increased levels may be correlated with CAD severity.

## Abstract (sr)

**Uvod:**

Predhodna ispitivanja pokazala su da su nivou serumskog vezujućeg proteina 4 (RBP4) povećani u metaboličkim poremećajima sa kardiovaskularnim oboljenjem (KVO). Međutim, može se reći da su ova izučavanja RBP4 u KVO oprečna. Iz tog razloga cilj je bio da se proceni odnos između RBP4 zavisno od pisustva i težine koronarnog arterijskog oboljenja oboljenja (KAO).

**Metode:**

U ovo izučavanje uključeno je 55 pacijenata sa akutnim koronarnim sindromom (AKS) i 43 kontrolne osobe sa različitim kardiovaskularnim faktorima rizika sa normalnom koronarnom arterijom pri koronarnoj angiografiji. Koncentracija RBP4 merena je primenom ELISA metode, a klinički i anatomski skorovi su korišćeni za procenu težine koronarnih lezija.

**Rezultati:**

Nivoi serumskog RBP4 bili su značajno viši kod pacijenata sa AKS u poređenju sa onima bez AKS (68,40 ± 47,94 mg/L vs. 49,46 ± 13,64 mg/L; p = 0,014). RBP4 je bio u korelaciji sa GENSI i SYNTAX I skorom (r = 0,034; r = 0,403; p = 0,002). Međutim, nije bilo odnosa između RBP4 i GRACE skora.

**Zaključak:**

Nivoi serumskog RBP4 povećani su u pacijenata sa KAO i njihovi povećani nivoi mogu biti u korelaciji sa težinom KAO.

## Introduction

Coronary artery disease (CAD) has high mortality and morbidity rate in the world and one of the main mechanisms responsible for its pathophysiology is inflammation [1]. Although most of the clinical studies have shown that cytokines are an important cornerstone in the development of atherosclerosis, the studies investigating the relationship between adipo kines with CAD have recently begun to find a place in the literature [2]. One of the interesting adipokines is the retinol binding protein 4 (RBP4) released by mature adipocytes and active macrophages [3]. RBP4 increases gluconeogenesis by stimulating phosphoenol pyruvate carboxylase and also lead to fat accumulation in visceral adipose tissue [4] [5].

There are many studies showing high RBP4 levels in obesity, hypertension and insulin resistance closely related with CAD [6] [7]. Recently, increased RBP4 levels were correlated with carotid intimamedia thickness and atheroma plaques circulation in patients with rheumatoid arthritis. Also another study confirmed that high RBP4 levels may indicate an increased risk of CAD in women [8] [6]. Although it has been claimed that RBP4 may have a role in the development of subclinical atherosclerosis, its role in the progression is not clear yet. This raises the debate whether increasing levels of RBP4 in the circulation would be a predictor for CAD. Therefore, we aimed to investigate the relationship between RBP4 levels with the presence and severity of CAD identified by angiograpically and clinically risk scores in patients presenting with acute coronary syndromes (ACS) in this study.

## Material and Methods

### Study population

This observational case-control study was carried out with 55 consecutive patients with ACS who admitted to the emergency department of Pamukkale University Faculty of Medicine Hospital, and 43 control subjects with normal coronary artery who underwent coronary angiography between January 2018 and June 2018. Inclusion criteria were the age range of 19-90 years, performing coronary angiography due to ACS or performing coronary angiography due to positive and suspected ischemia in non-invasive stress imaging tests in patients with at least one cardiovascular risk factor such as hypertension, diabetes or smoking. Previous myocardial infarction or CAD history, cardiomyopathies, severe heart valve diseases, acute pericarditis/myocarditis, cerebrovascular diseases, malignancies, hematological diseases, acute and chronic infections, chronic inflammation, autoimmune diseases, chronic renal failure (The calculated glomerular filtration rate using Cockcroft-Gault formula<60 mL/min/1.73 m^2^), severe liver disease were the exclusion criteria.

The acute myocardial infarction (AMI) was diagnosed on the basis of typical symptoms of myocardial ischemia (chest discomfort or angina equivalent), newly developed ischemic ST-T changes (ST-elevation or ST-segment depression or prominent T-wave inversion) in at least 2 contiguous ECG leads and elevated cardiac-associated biomarkers of necrosis in an appropriate clinical presentation.

This study was consistent with the Helsinki declaration and was approved by the hospital's ethical review board (Pamukkale University Faculty of Medicine Hospital, Denizli, Turkey). Written informed consent form was obtained from all subjects.

### Data Collection

Peripheral venous blood samples were collected after 8-12 hours fasting from all subjects. Routine biochemical parameters including fasting blood glucose, kidney function tests, lipid parameters and C-reactive protein (CRP) were analyzed with Cobas 8000 Autoanalyser (Roche Diagnostic Corp., Mann heim, Germany) and hematologic parameters were measured using Mindray BC-6800 Autoanalyser (Mindray Bio-Medical Electronics Co., Ltd., Shenz hen, China).

The serum concentration of RBP4 was measured using a commercially available enzyme-linked immunosorbent assay kit (YLbiont, YLA1249Hu, Shanghai YL Biotech Co., Ltd, China) in accordance with the manufacturer's instructions. Samples were diluted 2-fold into the dilution buffer provided. The detection limit of the assay was 0.26 mg/L (range: 0.5-180 mg/L). All samples were assayed in duplicate. The absorbance was measured at 450 nm with Biotek Elx800 microplate reader (BioTek Instruments Inc., USA). The data were processed with the Gen5 Data Analysis software (BioTek Instruments Inc., USA) and RBP4 results were calculated based on the standard curve generated by using a five-parameter curve fitting equation and multiplied by the sample dilution factor.

Transthoracic echocardiography was performed with Vivid 7 GE (General Electric Vingmed Ultrasound, Horten, Norway) in the left lateral decubitus position and left ventricular morphology and functions were evaluated. The left ventricular ejection fraction was calculated by using bi-planar Simpson method.

### Coronary Angiography and Coronary Lesion Severity Assessment

All patients underwent transfemoral angiography using the standard Judkins technique. Diagnostic angiograms were recorded using a digital media viewer and the records were reexamined by 2 experienced cardiologists who were blind to the study protocol.

Global Registry of Acute Coronary Events (GRACE) score including 8 parameters (age, heart failure, systolic blood pressure, creatinine, cardiac arrest at admission, ST segment deviation on ECG, abnormal cardiac enzymes and killip class) was used for the clinical risk assessment [9]. Anatomically coronary lesion severity was evaluated using SYNTAX I and GENSINI risk scores. The Gensini score was calculated by assigning a severity score to each coronary lesion according to the degree of narrowing and localization importance. The total score was equal to the sum of the stenosis score and the location score for all diseased segments [10]. SYNTAX I score was calculated for stenosis diameter of 50% or greater in vessels of 1.5 mm or more in diameter and the latest online updated version (2.11) was used (www.syntaxscore.com) [11].

### Statistical analysis

All data were analyzed using SPSS v.21.0 for Windows (SPSS, Inc., Chicago, Ill., USA). Categorical variables were expressed as frequencies and percentages; continuous variables were expressed as means ± SD. The normal distribution of the data was evaluated using the Kolmogorov-Smirnov test. The variables with normal distribution were compared using Student T test and Mann-Whitney U test was used when the normal distribution was not provided. Comparison of categorical variables was performed with chi-square test. The correlation between variables was tested via Spearman or Pearson correlation analysis and p <0.05 was considered as statistically significant.

## Results

The demographic-clinical characteristics and other findings of the study population are summarized in (Table 1).

**Table 1:** Baseline characteristics, laboratory findings and medications

Variables	ACS group (n=55)	Control group (n=43)	p
Mean age (years)	66.49 ± 12.27	57.00 ± 11.06	<0.001
Males, n (%)	34 (62)	17 (40)	0.028
Hypertension, n (%)	29 (53)	17 (40)	0.194
Diabetes mellitus, n (%)	22 (40)	13 (30)	0.317
Smoking, n (%)	12 (22)	8 (19)	0.695
LVEF (%)	47.22 ± 10.27	51.19 ± 18.08	0.174
FBG (mmol/L)	8.48 ± 4.06	7.32 ± 4.46	0.183
Creatinine (mmol/L)	85.75 ± 29.17	83.10 ± 19.45	0.661
TChol (mmol/L)	4.39 ± 0.87	4.03 ± 1.69	0.182
LDL-C (mmol/L)	2.59 ± 0.79	2.29 ± 1.12	0.132
HDL-C (mmol/L)	1.05 ± 0.25	1.13 ± 0.56	0.387
TG (mmol/L)	1.63 ± 0.91	1.74 ± 1.04	0.588
Hemoglobin (g/L)	126.3 ± 20.9	133.0 ± 14.6	0.075
WBC (×10^9^/L)	10.55 ± 4.15	9.74 ± 2.77	0.271
CRP (mg/L)	14 ± 31.3	10.2 ± 14.5	0.588
RBP4 (mg/L)	68.40 ± 47.94	49.46 ± 13.64	0.014
GRACE score	115.62 ± 24.24		
GENSINI score	66.41 ± 50.53		
SYNTAX I score	27.30 ± 9.73		

**Figure 1:**
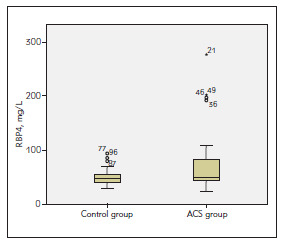
RBP4 concentrations in study population

The mean age and gender distribution differed among groups (66.49 ± 12.27 years vs. 57.00 ± 11.06 years, p<0.001; 62% (34) vs. 40%, [12], p=0.028). There were no differences in incidences of hypertension and diabetes, biochemical parameters including fasting blood glucose, creatinine, lipid parameters, complete blood counts and CRP (p>0.05). The serum concentration of RBP4 was higher in patients with CAD (68.40 ± 47.94 mg/L, 49.46 ± 13.64 mg/L; p=0.014) (Table 1, Figure 1).

**Figure 2:**
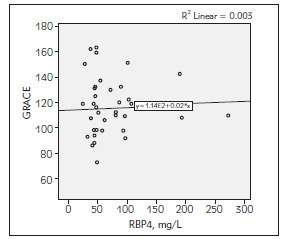
The correlation between RBP4 with GRACE score

**Figure 3:**
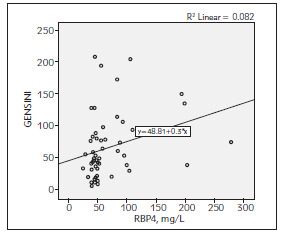
The correlation between RBP4 with GENSINI score

**Figure 4:**
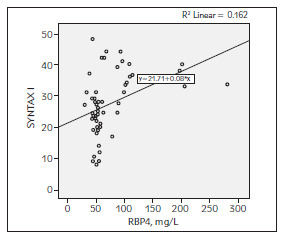
The correlation between RBP4 with SYNTAX I score

The average GRACE, GENSINI SYNTAX I scores were calculated as 115.62 ± 24.24, 66.41 ± 50.53 and 27.30 ± 9.73 respectively in patients with ACS. There was a positive correlation between RBP4 with GENSINI score (r = 0,286 and p = 0.034) and SYNTAX I score (r = 0.403 and p = 0.002). However, it was not found any relationship between RBP4 and GRACE risk score (Table 2, Figure 2, Figure 3, Figure 4).

**Table 2:** The correlation analysis of RBP4 and laboratory parameters

RBP4		
Variables	r	p
GENSINI	0.286	0.034
SYNTAX I	0.403	0.002
GRACE	0.050	0.768
LVEF	0.125	0.362
WBC	-0.174	0.205
Hemoglobin	0.051	0.71
FBG	0.081	0.558
Creatinine	-0.024	0.862
TChol	-0.015	0.916
LDL-C	0.018	0.898
HDL-C	0.065	0.637
TG	-0.102	0.457
CRP	-0.070	0.613

## Discussion

We evaluated the role of RBP4 in determining the presence and severity of CAD in this study. We found that the serum RBP4 levels were higher in ACS patients than in controls. Also GENSINI and SYNTAX I scores showed significantly correlation with RBP4, but not GRACE score.

Atherosclerosis usually develops with chronic vascular inflammation and one of the inflammation source is cellular oxidative stress. It has been suggested that RBP4 may be associated with oxidative stress and Farjo et al. confirmed that RBP4 initiates the process by stimulating gene expression associated with endothelial inflammation [13] [14]. But the studies investigating the role of RBP4 in vascular inflammation are conflicted [15]. In a study with a Saudi female population, serum RBP4 levels were higher in patients with CAD than in diabetic patients without CAD [16]. In another study, it was found 48% increased serum RBP4 concentration in female patients with CAD. Also in subgroup analysis of this study, the RBP4 levels were higher in ACS patients than in patients with stable angina pectoris and the SYNTAX score showed a positive significant correlation with RBP4 in both groups [7]. However, only the female gender was included in these studies. In a study involving both genders RBP4 levels were higher in patients with both ACS and stable angina pectoris compared to patients whose CAD was ruled out by coronary angiography. Although RBP4 was associated with high GENSINI score, RBP4 levels did not differ between CAD subgroups in this study. This status was explained as that RBP levels may be more affected by the presence or progression of CAD rather than its clinical presentation [17]. In our study, the increased RBP4 levels in patients with CAD and the relationship of RBP4 with GENSINI and SYNTAX I scores confirmed the results of these studies. However, we did not include patients with stable angina pectoris in our study. In addition, we did not show any correlation between RBP4 with GRACE score. It may be linked to the components of GRACE score including only clinical parameters instead of angiographical findings. Also the clinical symptoms and findings may not always overlap CAD severity and clinic presentation may differ in each patient due to genetic, age, gender, presence of comorbidity, current smoking, medications and early or late admission time to emergency department. Also the high number of hemodynamically stable patients, the collection of blood samples without waiting for troponin increases in patients with STEMI may have led to a lower average GRACE score, which may have caused its association with RBP4 to disappear in this study. Indeed, in a study with NSTEMI patients, a weak correlation was found between SYNTAX I and GRACE score, and it was reported that the GRACE score may indicate significant CAD disease, but it may not be appropriate to use for CAD severity assessment [12].

Unlike our study, some studies have failed to show relationship between CAD with RBP4. In Korean adults who underwent coronary angiography due to stable angina pectoris, the RBP4 level did not differ between patients with and without CAD [18]. However, the mean age of the subjects in this study was lower than our study, and ACS patients were not included. Moreover, the severity of CAD was evaluated using the number of diseased vessels and the number of patients with single-vessel disease were higher and the patients had lower serum RBP4 levels in this study. Another study found that serum RBP4 levels in patients with CAD were significantly lower than non-diabetic control subjects [19]. The heterogeneity of the study population, the cardiovascular risk exposure time or the methodological differences in measuring serum RBP4 levels may cause the different outcomes of the studies.

It has been shown that serum RBP4 levels increase with deterioration of renal function but we failed because of not including subjects with GFR<60 mL/min/1.73 m^2^ calculated by Cockcroft-Gault formula. It is expected that increased levels of RBP4 may affect lipid parameters that pose a risk for CVD [20]. However, we did not show any relationship between lipid parameters with RBP4 in this study unlike previous studies [21] [22]. Lack of adjustment for potential confounders, using of lipid lowering drugs, diet habits and heterogeneity in visceral fat distribution may have lead to this result. In addition, CRP which an inflammatory biomarker was not correlated with RBP4 in our study While most studies showed a positive correlation between CRP with RBP4, some studies failed to demonstrate the association between RBP4 with CRP when vitamin A status and dietary vitamin A intake were ruled out [23] [24].

The major limitation of our study was the cross-sectional design, which prevented us from inferring cause-effect relationship of RBP4 with CAD. Also we did not asses the long term clinical outcomes of increased serum RBP4 levels in ACS patients. This study had relatively small sample design and we could not rule out the effects of pharmaceutical agents on RBP4 levels because the majority of subjects with various cardiovascular risk factors were under treatment. The control group had high probability of CAD so we may not extrapolate our conclusions to healthy subjects. The groups were not matched for age and gender and this may affect serum plasma RBP 4 level. Finally, methodological differences in measuring serum RBP4 levels may change the outcomes of the studies.

Serum increased RBP4 levels may show the presence and severity of CAD. However, the large multi-center studies are needed to better determine the role of RBP4 in CAD and to evaluate its prognostic significance.

*Funding*: None.

## Conflict of interest statement

All the authors declare that they have no conflict of interest in this work.
